# “That Would Give the Kids a Little Challenge to the Brain!” Co-Investigating the Child Food Insecurity Experiences Scale (CFIES) with School Aged Children: A Qualitative Cognitive Interview Study

**DOI:** 10.3390/nu18142303

**Published:** 2026-07-14

**Authors:** Amanda J. Taylor, Helen Anna Vidgen, Sabine Baker, Danielle Gallegos

**Affiliations:** 1 Centre for Childhood Nutrition Research, Faculty of Health, Queensland University of Technology (QUT), 62 Graham Street, South Brisbane, Brisbane, QLD 4101, Australia; h.vidgen@qut.edu.au (H.A.V.); sabine.baker@qut.edu.au (S.B.); danielle.gallegos@qut.edu.au (D.G.); 2 School of Exercise and Nutrition Sciences, Faculty of Health, Queensland University of Technology (QUT), 149 Victoria Park Road, Kelvin Grove, Brisbane, QLD 4059, Australia; 3 Jacaranda Place Adolescent Extended Treatment Centre, Child and Youth Mental Health Service, Children’s Health Queensland Hospital and Health Service, Brisbane, QLD 4032, Australia

**Keywords:** child, food insecurity, qualitative, cognitive interviews, face validity, validation, assessment, measure

## Abstract

Background/Objectives: Household food insecurity (FI) impacts child health and development. Children have distinct and unique experiences of FI, and child self-report measures are increasingly used. However, there is limited reporting of children’s comprehension and engagement with these measures within local settings. The Child Food Insecurity Experiences Scale (CFIES) is a 10-item child self-report tool that is widely used, despite limited validity evidence. This study aimed to explore the face and content validity of the CFIES in an Australian context. Methods: We used cognitive interviews with 25 children aged 8–12 years to document CFIES comprehension. Data were analyzed using inductive and deductive approaches. Results: Children demonstrated awareness of FI (even when food secure themselves) and displayed sophisticated understanding of its social ramifications. While the overall perception of the tool was generally positive, we uncovered that not all key terms or emotion words were universally understood, potentially impacting validity. Similarly, conditional clauses or question modifiers were sometimes overlooked. In addition, the 12-month response timeframe and the answering categories (“many times”, “one or two times” or “never”) proved challenging for some children. In addition to the item-specific impressions, children provided nuanced ideation across four broader themes, which may impact item interpretation and willingness to answer: perceptions of hunger; micro and macroeconomics affecting households; social identity and comparison; and caregiver protection dynamics. Children indicated the need to consider survey modality and ensure privacy. Conclusions: A global tool that reflects children’s cross-cultural conceptualization of FI represents a pragmatic and feasible way to monitor change in FI across regions and may aid advocacy efforts. However, this study suggests the need for further modifications to the CFIES and the necessity to build rapport with communities and organizations that allow for the interpretation of the data within the contextual considerations.

## 1. Introduction

Food and nutrition security (FNS) exists when “all people at all times have physical, social and economic access to food, which is safe and consumed in sufficient quantity and quality to meet their dietary needs and food preferences allowing for a healthy and active life” [1]. Recent work on an updated definition of FNS for high-income countries demonstrated expert consensus on the inclusion of “culturally relevant foods” and the means of obtaining foods in “socially acceptable ways” as important conceptual additions [2]. Six interconnected dimensions are described as necessary to fulfil the definition of food security: food access, availability, utilization, stability, sustainability and agency [3]. Food insecurity (FI)—or the absence of any one of the above conditions—is a significant public health and social issue that seriously undermines optimal child health and development globally [4,5]. Understanding the consequences of FI for children and designing effective solutions to respond to FI in different contexts requires appropriate measurement approaches [2,6].

At the household level, the United States Department of Agriculture (USDA) Household Food Security Survey Module (HFSSM) [7] is the most deployed tool and has been used as the basis for measure development in other contexts [8,9,10]. Another tool, the eight-item Food Insecurity Experiences Scale (FIES) developed by the Food and Agricultural Organization, monitors FI prevalence globally and is used as an indicator of progress towards nutrition-related Sustainable Development Goals [11]. The HFSSM and FIES incorporate experience-based items relating to economic access to food. The HFSSM uses adult’s and children’s experiences in assessing the presence and severity of FI, while the FIES does not include children’s experiences. The HFSSM is often completed by the primary caregiver of a household due to their perceived central role in managing household food work and protecting children from the consequences of FI [12]. However, qualitative work with children has demonstrated that they experience FI differently from their caregivers and offer unique perspectives, which may be particularly salient in understanding the consequences of FI [13,14,15,16,17]. For example, a caregiver may report foregoing their own food to give their child as much they want, but that same child may intentionally consume less food without revealing their strategy [13]. Subsequently, there have been efforts to directly measure children’s perspectives of FI.

The development of child-specific measures aligns with the United Nations (UN) Rights of the Child (particularly Article 12), which positions children as having agency on matters that affect them, with the right to express their views freely [18]. Children have the right to be included in discussions and decisions about their lives, including via participation in research [18,19]. Incorporating children’s views directly aids the development of more effective policies, services, resources and interventions in tackling FI [20]. Considering children’s experiences of FI and its impact on children’s lives also supports the food security dimension of agency [21].

Pragmatically, measures that incorporate the lived experiences of their target respondents are reflective of best practice in measure development [22]. Face and content validity, which arguably underpin all other psychometric properties, are important yet underreported measurement properties [23,24]. Increasingly, consensus-based quality standards (for example COSMIN guidelines) include the degree of potential respondent involvement in conceptualizing measure constructs. These guidelines also consider whether a measure’s comprehensiveness, comprehensibility and relevance have been suitably tested and reported in the different contexts where it might be adapted for use [24].

A recent review identified and characterized child-report FI measures globally [25]. It identified a variety of child-report measures (ranging from one to 15 questions), and while use is increasing, many have not been specifically validated in their settings of use. The most used measure is the Child Food Security Survey Module (CFSSM), an adaptation of the HFSSM for children [26]. However, this nine-item measure is largely based on an adult conceptualization of FI and not children’s experiences and has been predominantly used in children aged 12 years and over. The review outlines four measures that extensively included children in their development and based their items on children’s conceptualizations of FI. These measures include the Child Food Security Assessment (CFSA) [27]; the Venezuelan Food Insecurity scale [28,29,30]; the Arab Child Food Security Scale (ACFSS) [31]; and the Child Food Insecurity Experiences Scale (CFIES) [32].

The benefits of using cognitive interviews to test the face and content validity of items have long been noted given that such testing can generate clearer understandings of what items are measuring from the perspective of respondents and can aid interpretation and analysis [33,34]. For the commonly used child-report measures, however, much of the face/content validity testing was conducted between ten and 20 years ago. Cognitive testing for the CFSA was undertaken in 2013 in South Carolina, United States, with 24 six-to-17-year-olds [27]. For the CFIES, a small group of Canadian children (N = 8) tested understanding and wording of the ten items in cognitive interviews. However, no further details were provided regarding how the items were tested nor the approach used to analyze the data [32]. Despite this, the CFIES is the most recently developed child-report FI measure that draws upon 20 years of research with children in this area and has been deployed via pilot survey in 13 economically diverse countries with children aged 5 to 18 years [25,32]. Given the CFIES is underpinned by the other child measures, including the CFSA, and that the CFIES is being positioned as a measure of choice to complement other FI indicators such as the FIES [32], it was chosen as the best measure to explore in an Australian setting.

This study aims to explore face and content validity of the CFIES in a novel Australian setting. It aimed to (1) establish children’s comprehension of the CFIES items, reference period and response categories, (2) identify any issues with the question sensitivity of the CFIES (extent to which questions are perceived to be personal or private), (3) identify if there are any other elements of the FI experience expressed by children that are not currently captured in the CFIES and (4) assess if the CFIES is fit-for-purpose for the Australian setting with children aged 8 to 12 years. This work contributes valuable face and content validity of the “globally applicable” CFIES for Australian children. The study employed participatory and child-centered qualitative approaches that work with children as “experiential experts” [35] and supports their rights to participation in decisions and discussion that affect their lives.

## 2. Materials and Methods

### 2.1. Study Design and Approach

This study employed qualitative semi-structured interviews, drawing upon cognitive interviewing techniques to assess the ten CFIES items (see Table 1). Initially, a detailed semi-structured interview guide incorporating verbal probing and “think aloud” techniques was created and reviewed by three experts in the field of qualitative work with children and/or cognitive interviewing. As ethical considerations were considered central to this study, the interview guide also included detailed procedures to ensure the safety of children, especially given the potentially sensitive topic. Additionally, pilot interviews (N = 8) were conducted with children known to the research team (with parental and child consent and assent) to test the proposed cognitive interview method and framing as well as recruitment materials and consent processes. Feedback from both children and experts was incorporated into the final interview guide.

We framed the interview approach as “detective work” and invited children to co-investigate questions about the household food situation with the research team. This specifically involved using study materials (participant information collateral, research onboarding discussions) to position the lead interviewer (AJT) as being highly interested in learning from children and joining with them to investigate the CFIES items together. Caregivers provided informed consent for their child to participate, and children provided their own independent informed consent and assent. Creative and participatory activities were incorporated into the study design to facilitate children’s participation.

The Consolidated Criteria for Reporting Qualitative Research (COREQ) checklist was used to guide study reporting [36]. See Supplementary Material File S1 for completed checklist.

Ethical approval was received by Queensland University of Technology Human Research Ethics Committee (approval no. 6749) and UnitingCare Queensland Human Research Ethics Committee (approval no. 202404).

### 2.2. Setting, Sample and Recruitment

Participants were eight-to-12-year-old-children who resided within a two-hour commute to Brisbane, the capital city of Queensland, Australia. The lower limit of the age range was chosen based on understanding that most eight-year-olds have developed cognitive capabilities to reliably complete self-report measures [37]. The upper limit of 12 years was chosen as this is a common cut-off point to delineate the start of adolescence, a period associated with greater autonomy including personal income generation and increased control over food choices [20,37]. Additional inclusion criteria required the child to be living at least part-time with a parent/caregiver who was willing to provide consent and caregiver agreement that the child was able to complete the research tasks (for example, speaking to a researcher for around 30 min about ten survey questions).

The study aimed to recruit for diversity across FI experiences, cultural backgrounds and household types to sample the varying circumstances that might be captured by the CFIES. The recruitment strategy encompassed multiple channels including email outreach to organizations working with children and families that shared the recruitment material with their clients. Organizations included those providing recreational services or sports activities to children, community youth groups, family support services, charities, churches providing emergency relief to families, food pantries and neighborhood community centers. Additionally, unpaid research notices were posted in several social media (Meta™ Facebook^®^, Menlo Park, CA, USA) community groups within the greater Southeast Queensland area. Participants were not known to the research team prior to data collection.

All recruitment materials contained QR links to a dedicated study website containing an “expression of interest” form and participant information in both child-friendly and caregiver formats. Interested participants could contact the research team directly or request to be contacted via their preferred method (call, text or email). Initial phone calls provided more information about the study and discussed potential preferences for interview location and time. After time for consideration, a second phone call confirmed interest and interview logistics and provided an opportunity for children to speak with the interviewer directly. Detailed disclosure and distress protocols were developed for the study, and aspects of these protocols were discussed with participants including limitations to confidentiality (disclosure of harm or suspected child maltreatment) or the ways in which the interviewer will be sensitive to any signs of participant discomfort.

Participating children were provided with a certificate of participation and $15 AUD cash to recognize their contribution. A $30 AUD EFTPOS gift voucher was provided to participating families to recognize the time and effort involved in coordinating research participation.

### 2.3. Data Collection

Face-to-face interviews were conducted between August 2024 and March 2025. Interviews were conducted by a single interviewer (AJT) who is assigned as “Mandy” in all quotes contained herein. Children chose their own pseudonyms for use in presenting the results. Children and caregivers negotiated the location of the interview within the family home or public space (for example, library) and the presence of the caregiver during the interview. Caregivers who were not involved in the interview remained nearby. The child’s choice of interview space was prioritized where possible.

Once settled in the chosen space, child consent was reconfirmed. Children could opt-out at any stage and could choose an alternative activity to complete in lieu of the interview. Stop signs of how to cease the interview or take a break were agreed with children prior to commencing, and children were invited to start audio recording when ready.

Generally, interviews followed the semi-structured interview guide starting with rapport-building and warm-up questions around the child’s favorite foods or exploration of additional materials in the “detective kit”. These included sensory toys, fidgets, drawing materials, stop signs, emoji images, and food photos included to facilitate the interview process and provide children a range of ways to feel comfortable or provide their responses. Each CFIES item, reference timeframe (In the last 12 months…) and response options (many times, 1 or 2 times, never) were printed and displayed on laminated cards in the interview space. To facilitate their agency, children could elect to have the item read to them by the interviewer or to read it independently on first view of the item. In practice, the interviewer read each question to the child at least once as part of the interview process.

Although the original interview guide included both think-aloud prompts and verbal probing approaches, the difficulty of some children to enact the “think aloud” approach in initial interviews resulted in a focus on verbal probing of children’s responses per item. Nevertheless, some children spontaneously commented on their thought processes in responding to the CFIES items. At the end of the interview, children were invited to share their thoughts on what it was like to take part. Caregivers completed a brief sociodemographic survey and the USDA 18-item HFSSM. They also received an information sheet detailing local wellbeing and food support services. A formal check-in procedure was enacted, with the interviewer contacting all families two days post-interview to answer any further questions or address any potential participant discomfort.

Research memos were written post-interview to document initial interview impressions, capture any key issues that arose and note any additional caregiver-provided information. Data saturation was considered as adequately achieved when later interviews tended toward informational redundancy and contained repeat comments from earlier interviews [38].

### 2.4. Data Analysis

Interviews were transcribed verbatim using Descript (Descript Inc., available from www.descript.com), an online automated transcription program. The first author (AJT) verified and cleaned interview transcripts via comparison to raw audio. Cleaned transcripts were imported into NVivo (QSR International, Release 14.23.1, available at https://lumivero.com/products/nvivo/, accessed on 1 July 2025). Transcripts were not returned to participants for member checking to reduce participant burden and support confidentiality in the case of children who did not want a caregiver physically present for their interview.

Initially, open line-by-line coding was carried out by all four authors on three transcripts. From this and initial in-depth discussion of codes between the team, a preliminary codebook was collated. Remaining transcripts were coded by one author (AJT) using line-by-line coding via both an open inductive lens, as well as a deductive lens influenced by relevant cognitive interviewing frameworks (see below). A project codebook containing coding clusters and themes based on the dual coding approaches was iteratively discussed and refined with the team. Regular, in-depth debriefing sessions between the entire author team across the data analysis process allowed for reflexive discussion, resolution of any disagreements and consensus building around the chosen analytic approaches. Preliminary themes from the inductive coding approach were seen as providing important context for the findings obtained via the deductive lens. Data was further interrogated to more deeply explore and describe dimensions of participant’s understanding and response to the CFIES using a cognitive interviewing coding framework from the literature (see below).

### 2.5. Cognitive Interviewing Frameworks

In the absence of child-specific frameworks, cognitive interviewing with children commonly adapts frameworks used with adults, while also recognizing children’s own unique developing capacities [39]. Three frameworks from the cognitive interviewing literature were influential in overall study design as well as the analysis process. Scott et al.’s [39] methodological paper regarding cognitive interviewing practices in global public health detailed a range of different survey tool components that may be assessed within a cognitive interview, including word choice; syntax; question sensitivity (extent to which questions may be viewed as personal or embarrassing); memory; response options; and resonance with local world views and realities. These survey components were used as the basis for designing verbal probes and prompts around each of the ten CFIES items (see Supplementary Materials Table S2 for example probes using each of these components). These components were also used to deductively code children’s overall interpretation and understanding of the CFIES tool.

Underpinning Scott et al.’s [40] approach is Tourangeau’s (1984) classic cognitive model of the survey response process, which is described as involving the following steps: (1) comprehension (understanding question intent and meaning of terms); (2) retrieval (recallability of relevant information for the question and strategies to recall); (3) judgement (motivation to complete items, sensitivity/social desirability factors); and (4) response process (matching internal generated answer to response categories) [41].

This cognitive viewpoint can be further enhanced by overlaying a sociological cognitive lens, as advanced by Brekhus (2007), which recognizes cognitive response processes as inextricably shaped by cultural phenomena [42]. The six processes of perception, attention/inattention, memory/time/chronology, classification, meaning making and social identity were considered when re-examining preliminary themes constructed from open coding to help understand key sociocultural and contextual considerations surrounding children’s survey response processes.

### 2.6. Positionality Statement

All authors are white, cis-female; three are dietitian-nutritionists, and one is a psychologist. All have experience in frontline service delivery and research. The professional backgrounds of the author team were not highlighted in participant information and consent materials or in research onboarding discussions to foreground the research project’s focus on children’s views of the CFIES items and to reduce any perceived power differentials between researcher/child/family. Two authors have extensive experience in FI, and one has extensive experience in measure development. All authors are parents with children of varying ages. The first author is a mother to a young child and infant; the third author is mother to school-aged children; and the second and fourth authors are the mothers to young adults.

## 3. Results

### 3.1. Characteristics of Participants

A total of 25 children participated from 17 families. Age, sex and sociodemographic characteristics of the children and caregivers are detailed in Table 2. Mean age of participating children was 9.6 years (range: 8 to 12 years) with a mostly even split between boys and girls. Around one-fifth (19%) of the children had a diagnosed health condition that could potentially affect eating or appetite, either directly or via its treatment (medications). Approximately one-fifth (19%) of children had caregiver-reported concerns relating to development, including social, emotional, developmental and learning delays.

Participating families came from a range of self-identified cultural backgrounds including Australian, Anglo-Australian/Italian, Asian, Vietnamese, British/Australian, Colombian, Iranian, Samoan/Australian, Russian, Jewish and Caucasian. Caregiver-reported FI as measured by the USDA HFSSM was identified in five households (33%), with any level of child FI reported in four households (Table 3). The items affirmed for child-level FI included those related to relying on a “few types of low cost” foods to feed children and being unable to feed children “healthy meals because there wasn’t enough money”. For adult and child-referenced items from the HFSSM, these refer to all adults and children in the household and not individual children’s experiences.

### 3.2. Characteristics of Participation

A total of 23 interviews were conducted. No children or caregivers opted to drop out within or after the physical interview process, including after confirmation of consent/assent with children. All interviews were face-to-face, audio-recorded and lasted between 9 and 67 min (mean length = 31 min). Eight sibling pairs were interviewed. Two elected to participate together in one interview. Six chose to participate in separate individual interviews.

Seventeen caregivers were involved in the study (all providing informed consent for children to participate and 15 completing the sociodemographic survey). However, the degree of caregiver involvement in each individual interview varied as children were given choice over who they wanted to be present for the interview. Caregiver involvement was categorized as follows: nil: not present in same immediate space as interview but may have been nearby (e.g., cooking in kitchen in next room) (*n* = 10); involved—minimal: caregiver present in interview setting but did not contribute or intervene in interview (working on laptop at table in same room with headphones) (*n* = 6); involved—active: caregiver present in interview setting and provided extensive support, prompting or content in course of interview (*n* = 7). All actively involved caregivers were present due to expressed wishes of participating children. All caregivers present at the time of interview were mothers apart from one, where the caregiver present was an adult older sister (the child’s mother provided informed consent to take part in the research).

#### 3.2.1. Caregiver–Child Dynamics in Interview Space

Typical interactions between children and their caregivers are described below. Some caregivers assisted their children with interview tasks, on request of the child: “Can you please read it for me? [referring to CFIES item one]” [request directed to Sophia’s mum] (Sophia, 8 F). Or as seen as this in exchange between Zac (8 M) and his mother, discussing the food experiences of another family:

Zac: “But don’t, don’t they not, not even know about the two different families?

Zac’s mum: “They don’t know yet. But, um, I mean like they eat different things when they go to their dad’s house and when they go to their mum’s house, they have two different kinds of standards of food that they eat.”

Zac: Yeah.

Zac’s mum: And they’re happier at one, more happy at one place than the other. Do you know what I mean?

Zac: *nods*”

For Tom (9 M) with English as a second language, there was significant back-and forth with his mother in both Russian and English when discussing and clarifying questions and responses:

Mandy: Let’s have a look at question four now. So this one is, “has the size of your meal been limited because your family didn’t have enough food?”

Tom: Can you say it again?

Mandy: Absolutely. Has the size of your meal been limited because your family didn’t have enough food?

Tom’s mum: *speaks to Tom in Russian*

Tom: Oh, yeah, that’s happened. That happened to me, like, lots of times when I was in Russia, because Issac was like, this, this, this, this, this, I want this, this, this, this, this. I only had, like, a soup left.

Mandy: Oh, wow. So…

Tom: *speaks in Russian to Mum*

Tom’s mum: He talks about that, uh, when they lived with grandma. The younger kid had everything. And he had only soup left. But the question is “has the size of your meal been limited because of your family didn’t have enough food?” *speaks in Russian to Tom*

Tom: Sometimes. Sometimes it changes, sometimes it doesn’t.

There were instances where caregivers prompted their children to answer: “Mum: Ace, did you hear what she said? Ace: What? Mum: What makes it hard?” (Ace, 10 M). Sometimes this prompting triggered children’s memories for relevant content:

Mum: What about some stories you guys have told me about your friends at school? When they come to school and you’re having lunch together?

Peppa Pig (11 F): Oh yeah, um, sometimes my, one of my friends doesn’t really pack much in her lunchbox.

Zac (8 M): Sometimes, um, my friend doesn’t have a lunch at all.

Caregivers at times also expanded upon or explained their children’s responses:

Archer (9 M): Nah. Um, I’ve been hungry, but haven’t eaten because she doesn’t have the food I want. Like, she doesn’t have any good crackers, or any good chips or anything.

Archer’s Mum: But there’s always some food, but not always the food you want?

Some children also displayed attunement to caregiver facial cues in the interview space: “Are you mad at me?” (Bethany, 11 F); “Um, are you about to cry?” [to mum] (Sophia, 8 F) and sometimes asked permission from caregivers to share information:

Sophia: I felt sad because I, I didn’t, I didn’t, am I…allowed to say it to mummy?

Sophia’s Mum: Yes sweetheart.

Sophia: Yes. I didn’t really like being out in the sun all day, but I knew it was the best to help mummy, so I wanted to do it.

Other children provided reassurance to caregivers:

Mandy: Did you worry about how hard it is for your parents to get enough food for your family?

Zac: I didn’t worry. *moves to hug his mum*

Mandy: You didn’t worry, sure…

Zac: *hugs his mum*

Mum: That’s so cute…

The potential “social desirability” impacts of caregiver presence were highlighted by some caregivers: “I was worried just then that they would be worried of me hearing what they had to say… Now the kids are getting older, they have this massive empathy. And like, if they think that they’re stressing me out. And I have to tell them, it’s okay, you can tell me. It’s just interesting to know.” (Mother of Peppa Pig (11 F) and Zac (8 M)). Peppa Pig and Zac felt their responses were not significantly influenced though this exchange itself may have been influenced by caregiver presence: “Mum: Do you think you guys would have said the same thing if mummy wasn’t here? Peppa Pig: Yes. Zac: Yes. Yeah.”.

Overall, these interview dynamics illustrate caregivers were generally seen as a trusted support person who children spontaneously called upon for help and assistance in the response process, who engaged in exchanges clarifying meaning and memory with their children but who also could prompt or scaffold children to provide further information, which may not have been offered for various reasons. The impacts of socially desirable responding when a caregiver is present are important to consider when contemplating survey administration.

#### 3.2.2. Child Actions in Interview Space

Children used their surroundings and props (supplied in the form of a “detective kit” with sensory toys/fidgets, emoji prompts, stop signs, art supplies, etc.) to help respond to interview questions. Some children spontaneously used props to help demonstrate their meaning. For Viper (9 M), who mostly used non-verbal body language to respond throughout the interview, he spontaneously used the emoji prompt sheet in discussing the term “family”:

Mandy: And what about the word family? Can you tell me some other words for family?

Viper: *points to emoji with big smile and love heart eyes*

Some children used the props to model emotional responses:

Mandy: So what if like, if Bunny was doing this questionnaire and Bunny was answering these questions and he said “many times” he felt embarrassed or ashamed ’cause his family didn’t have enough food…

Sophia: That’s okay, Bunny. It is okay. You’re okay *cuddling Bunny toy*. Yeah. At least you have money now. You live with a nice family.

The use of props and the building of trust between interviewer and child means that the face-to-face modality may offer potentially richer data than a self-administered setting: “Uh, I think it’s better if they did it face to face. Because then they’ll get all the other stuff as well… Uh, like, if you weren’t here then maybe I wouldn’t have said a story or something like that. And that also helps with the research.” (Kevin, 9 M). Others, however, noted that a self-administered setting might increase disclosure of information:”… sometimes speaking about emotions is hard…And I like sit there and I don’t know how to make eye contact so that, that could be a possibility, that’s why writing it out might help?” (Romie, 12 F).

### 3.3. Children’s Comprehension and Understanding of CFIES

This section explores how children comprehended and interpreted the ten CFIES items (Table 1), including key terms and suggestions for improved wording; children’s ability to use the response categories and 12-month response timeframe, and factors that may impact on children’s willingness to respond.

#### 3.3.1. Key Descriptive Terms

Table 4 outlines the main terms from the CFIES items that were probed with children within the interview. Verbal probes aimed to investigate children’s understanding of key words used in survey questions, rule out unintended meanings or overly vague or specific words and identify the most natural words to use in context.

The term “family” features in CFIES item one and two. Most children identified “family” as those who live with them, care for them and love them with synonyms suggested by children presented in Table 4. Similarly, “parents/guardians” were seen by children as those who protect them, look after them, feed them, take care of them, raise them and live with them. The additional inclusion of the term “guardian” did pose some difficulty for a few children, “What’s guardians?” (Tom, 9 M), and others felt younger children might struggle with the term.

The term “run out of food” also features in CFIES item one. Children were probed about how to tell if food had run out for a family and potential reasons for running out of food. Some children felt this could be identified through the contents of the fridges/pantries, others described behaviors or physical sensations. Children identified a wide variety of reasons why food might run out for families. These included due to resource constraints/economic access including low income and high food prices. Other reasons provided by children included impacts on food availability from natural disasters (Noting that Brisbane and south-east Queensland where this research took place had experienced major flooding events every year since 2020, with the most recent in March 2025, and at least one major bushfire in the last three years). Some children provided potential issues with housing as a contributor: “If the house isn’t protected enough, there might be rats… eating into the boxes!” (Strawberry, 9 F). Though as the exchange between Strawberry and her sibling demonstrates, “pests” might well include other family members eating more food than others:

Mandy: So, there might be rats eating into the boxes?

Ace: *laughs* Jacky!

Strawberry: *laughing*

Ace: A rat!

Mum: That’s his older brother.

Mandy: Oh, and so does he eat some of the food as well?

Ace: He eats a lot of food. He eats a lot of food.

Mandy: So other family members, that could be a reason?

Strawberry: Yep. Especially like Scott!

Mum: Scott. She’s got an older brother as well.

Other children also cited consumption of food as a reason for it running out generally, “Eating it” (Viper, 9 M), but also in the context of a meal occasion: “Um, maybe if you got too much or if you didn’t make enough…And then everyone else has got like little pieces.” (Scarlett, 9 F).

The term “limited” as included in CFIES item four was seen as potentially difficult to explain, “Like that would give the kids like a little challenge to the brain!” (Justin, 8 M), or to think of alternative words “Like, I had it now, I forgot it. Uh, just like, like, Uh, like, no…” (Peach, 12 F). Other children did use novel ways to explain the term, often using interview props or the surroundings to help demonstrate meaning: “Like, say this, um, instead of having this, you could only have one, and I’m able to have the rest of the things.” *Uses props (felt pens) from detective kit to demonstrate* (Sundew, 9 F). As displayed in Table 4, most children defined “limited” as referring to restricted quantity and a smaller amount. Upon probing who would limit the size of a meal, some children identified both a child and a parent as potential candidates: “Sometimes it could be a parent. Sometimes maybe it could be the kid if they want to help their parents.” (Sundew, 9 F); “Maybe if they (children) like understood that food was tight and so they might eat a bit less. But I don’t, like, that’s really the only situation I think they would do that.” (Bart, 12 M). Others identified only parents/caregivers as those who would limit: “Um, probably the parents because they’re worried they’re not gonna have enough food…because I’m pretty sure the kids would just eat.” (Bob, 12 M). Others were not sure who would limit meal size if there was not enough food at home: “I’m not sure. Maybe.” (Archer, 9 M). 

The term “skip a meal” also posed some complexities for children. Some expressed confusion. Most children recognized the term to mean not eating at a specific time (citing a meal occasion like breakfast, lunch or dinner) and provided a variety of reasons for doing this. One child with FI experiences explained they would say they were not hungry to skip a meal.

#### 3.3.2. Key Emotion Words

The CFIES contains several adjectives used to describe an emotional response to food insecurity. In items one and two this is a single word; that is, worry. In items seven to ten, two adjectives are given; those are, tired or weak, embarrassed or ashamed, and sad or mad. Table 5 provides a summary of the key emotion words explored with children, including alternative words, meanings and representative emojis. There was a tendency for children to spontaneously demonstrate emotions using their bodies and facial expressions: “You can tell if they’re tired by like, yawning, like, like that” *demonstrates yawning with eyes closed* (Justin, 8 M). This meant children felt most emotions were recognizable in others, though some children felt less able to identify such emotions in others, “Not many times” (Bob, 12 M), though children also recognized that emotions might be hidden from others: “You can’t always tell, but sometimes you can. It depends on how they’re acting. But sometimes people are really good at hiding it.” (Romie, 12 F).

For each of the items containing emotion words, these were often delivered “double-barreled” with two emotions presented. While there was evidence that many of the emotions were interchangeable, especially for words like worry/ashamed/embarrassed, some children also noted there could be nuance in expressed emotions making the double-barreled presentation potentially confusing: “They mean different things because you could be sad or you could be mad.” (Sundew, 9 F). Nonetheless, the fact that similar emojis were chosen to represent multiple emotion words suggests “emotional awareness of FI” may be of more relevance to children than emotional nuances.

Barriers to understanding as cited by children included issues with understanding key words and terms, as discussed in the previous section, “Because there’s emotions that you might not be familiar with.” (Goku, 9 M), particularly for younger children: “Um, someone might not understand the word weak or tired if they’re a really young audience, but I thought it was pretty straightforward.” (Romie, 12 F).

#### 3.3.3. Children’s Feedback on CFIES Item Structure and Alternative Wording

Though many children identified each of the items as “easy” to understand, upon further probing, issues with comprehension/interpretation were identified with some specific items, namely items two and 10. One of the main factors contributing to difficulty with understanding was the inclusion of multiple elements in the item: [Referring to item 10] “Extra reading does make it a bit harder.” (Starfire, 9 F); and

[Referring to item 2] “I’m not sure. Well, it’s more like…*reading question to self* I feel like the “how hard it is for your parents/guardians to get enough food for your family”. Kind of like made me think like, wait, what? ‘Cause at first I thought, does that just mean like, like if your parents or whoever takes care of you can get enough food? But I think cause like the ‘worry’ and ‘how hard it is’ kind of like made me think, wait what am I supposed to be like answering?”

(Bart, 12 M)

Shorter question length also seemed to relate to perceived ease of understanding: [Referring to item six] “Uh, I think it’s definitely one of the easiest because it’s very short.” (Peach, 12 F).

Table 6 details alternative wording provided by children for four specific CFIES items (1, 2, 4 and 10). Most suggestions involved shortening/cutting out words in the item, though this sometimes meant that the intended concept/FI domain may not have been captured.

#### 3.3.4. Children’s Understanding of 12-Month Reference Timeframe

Children appeared to be able to understand the reference timeframe of 12 months but did not apply this to their responses regarding being able to recall events specific to that timeframe. There was some evidence in the interviews that children struggled to use the 12-month reference period to frame their responses. Children generally defined 12 months as meaning “one year” (Justin, 8 M); “like over this year, and a bit of last year” (Freya, 10 F); “like in the last year” (Lily, 9 F). Other children offered alternative explanations such as “in like, the last two years, maybe.” (Zac, 8 M) or expressed uncertainty about the meaning of the 12-month period both verbally and non-verbally: “I don’t know.” (Bethany, 11 F); *shrugs shoulders* (Viper, 9 M).

Other children offered that such a time span presented a challenge to think about: “Way too long ago!” (Justin, 8 M), “Reaaaaally long!” (Sundew, 9 F), or as Starfire (9 F) expressed in relation to thinking about food, “It’s a bit hard like because it’s hard to think about what we’ve eaten in the last 12 months.”

For children who had FI experiences, there was a tendency to ignore the 12-month timeframe and think back to the relevant period, as can be seen in the below exchanges with Bethany (11 F), Ruby (8 F) and Romie (12 F):

Mandy: Okay. So did you worry that food at home would run out before your family was able to get more?

Bethany: Ummm…would that have been when we lived in [suburb]?

Mandy: So, in the, was that in the last 12 months?

Bethany: I can’t remember. Because if it was in [suburb]. Yeah. Maybe a few, one or two times.

Mandy: So “did you worry that food at home would run out before your family was able to get more?”

Ruby: When I was younger.

Mandy: When you were younger?

Ruby: Yes.

Mandy: Sure. So what about in the last 12 months? Is that hard to think of?

Ruby: Uh, I didn’t really think of that.

Romie: I remember when I…I remember when I was younger my mum didn’t eat sometimes so that she could afford for me to eat. So, that’s kind of how I would phrase it.

Mandy: Wow. So you really remember that?

Romie: Yeah.

Mandy: And was that like a long time ago?

Romie: Uh, well, considering I’m 12, it happened when I was like 6 or 7, so…

Mandy: And, but you sort of still remember that?

Romie: Frankly, yeah.

Children offered a range of methods of how they remembered 12 months/one year, such as using the standard calendar months (“I would just think of January to December because that’s the way everybody learns.” Bob, 12 M). Other children used significant events and holidays to remember (“Like, uh, I think a year is too long. So when, ‘cause when we don’t get enough Christmases, if the years were shorter, we would get more Halloween and Easters, and holidays!” Kevin, 9 M); “Um, like in the last year, what’s been happening, um, like what’s happened to me, what’s something big that’s happened?” (Mikey, 10 M). Others described the 12-month timeframe as being related to a place/location:

Mandy: When you hear the words “in the last 12 months”, what do you think of?

Starfire (9 F): Ummm…my two old houses that I used to live in.

Goku (9 M): Um, well that was last year, I recently moved houses and moved from the [area in north of state] to [current region].

Other children provided detailed description of how the passing of time can “feel” different, depending on the reference point.

“So whenever your mom and dad says, “Oh, you’re having a sleepover with your friend in two months” and you’re like, “Oh my God, that’s a long time away!”. But if you don’t like to go to the dentist and they say you’re going to the dentist in 12 months. “I don’t want to go to the dentist at all!” You just, it’s like 12 months, it’s like in two days! But in 12 months when we sleepover, you’re just like, when is it going to be 12 months? You’re just like, “Come on! I want to have a sleepover!”

(Scarlett, 9 F)

Overall, while children generally considered “in the last 12 months” as referring to the past year, there was evidence this was not clear for all children. Moreover, children with FI experiences outside of this timeframe did want to speak about them in this interview setting. Children also discussed some of the ways they think about a year and time more generally, and these contributions may provide insight into how reference time periods may be appropriately contextualized to facilitate their response.

#### 3.3.5. Children’s Understanding of Response Categories

There are three standard response categories provided for children to select from in answering the CFIES items: “many times”, “one or two times” or “never”. Despite having the possible response options displayed on a laminated card in front of them, children often elected not to use these categories when providing a response. Many responded with a simple binary yes/no answer: “No” (Bob, 12 M, response to item 4); “Nup” (Kevin, 9 M, response to item 1); “I have not, no” (Bart, 12 M, response to item 8); “Yes” (Justin, 8 M, response to Item 7).

Some children offered an elaborated answer that demonstrated their thought process behind the response: “Well, besides eating my meal, sort of, usually I have enough food in my meal, and whenever I don’t have enough, my parents give me more, like, for example, like, a piece of bread if I feel really hungry, or something like that. So, um, never, I think.” (Mikey, 10 M, response to item 4); “The only time I would skip a meal if I was not hungry and I ate way too much food for afternoon tea. But no, not because they didn’t have enough food.” (Pilot, 12 M, response to item 6).

When directly probed on how children perceived the different response options, a variety of meanings were offered. For the term “many times” in a period of 12 months, this varied significantly between children: “Well, at least, I don’t know, more than three.” (Goku, 9 M); “maybe about three or four…at least…or maybe many could be six or seven” (Sundew 12 F); “maybe like once a month…twice a month” (Peach, 12 F); “20” (Pilot, 11 M); “Something would happen like every week.” (Freya, 10 F); “Like about like 100 to like 500.” (Justin, 8 M); “It’s just a lot. I understand it’s a lot.” (Mikey, 10 M).

An exchange with Ruby (8 F) demonstrated her thoughts about the three response options and indicates that children could potentially interpret the response options quite differently than intended:

Mandy: So how many times would something have to, to happen in a year to be many times to you?

Ruby: Like every day?

Mandy: So something would happen every day and that would make it many times?

Ruby: *nodding*

Mandy: Great. And one or two times over a year?

Ruby: Maybe. Just like sometimes in a day.

Mandy: Sure. Great. And then never? The word ‘never’?

Ruby: Maybe we just forgot about that.

The uncertainty of memory/recall is further illustrated by Sophia’s (9 F) response to item four, “size of meal being limited”. After requiring a further explanation of the question meaning by both the interviewer and her present caregiver, she offered “I don’t know. I can’t remember from when we didn’t have much money”. Pilot’s (11 M) offering of alternative response options attempts to simplify wording but also recognizes the uncertainty that children may display according to their experiences: “Um, actually maybe like “yes”, “maybe” and “no”….yeah, because they’re just really easy words that we’ve all like that we’ve learned.”

### 3.4. Children’s Perceptions of the Question Sensitivity of CFIES

Children were also probed regarding if they felt discomfort in the process of answering an item or if they thought other children might. Many children expressed ambivalence about item sensitivity, with most denying any individual discomfort for each of the specific items but recognizing the potential for discomfort for other children, particularly if they had FI experiences: “Some kids might, but some kids won’t. Different, different.” (Strawberry, 9 F); “No. Yes. Maybe. I don’t know.” (Bethany, 11 F); “I think it could be uncomfortable for some kids that don’t really have enough money, or their parents don’t have enough money, but for people that, like, get pretty much good and enough food, it not really that embarrassing. It’s more like a bit okay to answer.” (Lily, 9 F). Other factors influencing whether a child found a question uncomfortable was if they struggled to understand the question intent or were uncertain about their answer: “Because they don’t, like, know the meanings to it.” (Strawberry, 9 F); “Maybe, um, if you, if you don’t know or, you don’t want to say it out loud?” (Peppa Pig, 11 F). Mikey (10 M) offered an alternative perspective that children may feel guilty responding a certain way due to not having to skip meals due to not enough food: “Because maybe they don’t want to answer. And because they feel bad for other kids who can’t get full? I feel sort of the teensiest bit guilty, but because I’m talking to one person, not really.”

Other children felt specific items tapped into private matters, “I don’t want to tell anybody else about it.” (Sophia, 9 F), and their responses could negatively implicate their caregivers: “[Referring to CFIES item two] A bit embarrassing because like, that means like their parents doesn’t have enough money.” (Lily, 9 F); “[CFIES item two could be embarrassing] maybe if their parents didn’t have jobs?” (Bob, 12 M).

### 3.5. Further Contextual Considerations

In addition to the item-specific responses explored above, children also provided nuanced ideation about other deeper concepts such as perceptions of hunger; micro and macroeconomics affecting households; social identity and social comparison; and caregiver protection dynamics. In doing so, further illuminating insights into how children view the process of talking about FI more generally and how they conceptualize food in their wider environment were revealed, with implications for the response process.

#### 3.5.1. Perceptions of Hunger

Across interviews, hunger was often discussed as a highly salient physiological state that was unpleasant, “I used to feel hungry and it was scary” (Bethany, 11 F); “My tummy will start to hurt. And if I don’t listen to my tummy, my brain will start to hurt” (Sophia, 8 F); as well as visible and audible: “I mean, your stomach’s rumbling. You can kind of feel that you’re a bit like, “Awwww!”. You can kind of feel that way” *holds stomach*” (Bart, 12 M). The state of hunger was also linked to feelings of anger: “Um, like if I’m, if I’m hungry, like my stomach starts rumbling and I get hangry…I just get frustrated because I just want food.” (Peach, 12 F) with the concept of “hangry” being highlighted by multiple children in both themselves and others: “Well, I do get hangry if I don’t eat…*points to mum* She gets even more hangry when she doesn’t eat.” (Archer, 9 M). The high salience of hunger to children meant in some instances children linked emotional concepts in the CFIES items to physiological hunger, versus these emotions being linked to feelings of deprivation due to a lack of enough food:

Mandy: Did you feel sad or mad because your family didn’t have enough food?

Bart: Um, no. I mean unless you’re like saying getting hangry or something.

Mandy: Why might someone feel sad or mad if their family didn’t have enough food?

Kevin: Oh, they might feel um hangry.

Being hungry as consequence of not having enough food within specific short-term timeframes was also discussed: “Maybe after like a soccer game. And then like, I have breakfast and then I have soccer. And then we might have to do something else. Go straight in the car to go somewhere.” (Mikey, 10 M); “I mean, I’m always hungry when…I’ve eaten all my lunch at first break.” (Archer, 9 M). Being hungry for certain types of foods also was mentioned by many children: “Like there’s, there’s always options. I just don’t want to eat an apple.” (Peach, 12 F). Other impacts on physiological hunger included distraction when playing video games: “It usually doesn’t come to mind. And then I just come off the computer, I’m like, “Oh, I haven’t had food in a long time!…It’s like as soon as I get off and I am bored, I feel it so immediately” (Pilot, 11 M). Medications could have a suppressant effect on appetite, “my medication makes me not hungry” (Pilot, 11 M), or heighten appetite, “No, I used to feel hungry and it was scary…that was recently, it was when I was on the medicine.” (Bethany, 11 F). Overall, children’s sophisticated discussion of different aspects of hunger should be accounted for in considering their response processes to key CFIES items, particularly items five, seven and nine. As shown in these exchanges with Pilot and Mikey, the “not enough food” modifier in the item was seemingly not accounted for in their responses to item seven:

Mandy: So, “did you feel tired or weak because your family didn’t have enough food to eat?”

Pilot: I have only felt tired or weak if I don’t have the food myself because I don’t usually have food during the day as my medication makes me not hungry. The only time is if my body just shuts down because I never knew that I actually needed to eat.

Mandy: So “did you feel tired or weak because your family didn’t have enough food to eat?”

Mikey: I think whenever I don’t eat food, I feel, I do feel tired. I do feel, I sort of feel a bit like sick. My mouth like goes dry. And I feel like I really need something to eat. And yeah, I’m just tired.

#### 3.5.2. Perceptions of the Household Food Situation—Micro- and Macroeconomic Factors

Children also displayed sophisticated understanding of the micro- and macroeconomic factors impacting their household’s food situation. Children displayed significant awareness of rising living costs in their macroenvironment: “‘Cause usually the prices was low before and now it just go higher and higher by the day” (Justin, 8 M); “Literally everything is so expensive. Like, I just wanted to buy a pack of gum, and it’s like $3…What happened to when it used to be like 50 cents or a dollar?” (Romie, 12 F).

Children discussed the various budgeting and economizing behaviors within their household motivated by these economic realities: “Probably, we had to shop at Aldi because like, we just bought a house and we kind of needed a bit more money so we just shopped at the more cheaper shop.” (Lily, 9 F). Children were aware of bulk buying practices or buying unbranded products, “Oh, our dad stocks up at Aldi…and he gets a lot of chocolate…like three kilos.” (Kevin, 9 M), as well as altering purchases to seasonal patterns, “We stopped eating grapes because it’s just too much now…Like a little bit of grapes, it’s like $12 or something like that.” (Scarlett, 8 F). Other intrahousehold strategies around food management were noted:

“We normally have like leftovers for dinner. And also sometimes we don’t eat all of it. And sometimes mum tells dad to stop eating the kids’ food. Because he eats it, like, “save it for seconds!”

(Lily, 9 F)

“Dad, can I have strawberries?…He said yes, but only one because the other ones are for your lunchbox at school.”

(Scarlett, 8 F)

The awareness of these strategies by children in their environments may influence how they comprehend, retrieve and interpret their experiences in relation to the CFIES items. This is particularly so for item 3, “not able to get foods you wanted due to not enough money”. While this item intends to represent children’s lack of choice and feelings of deprivation due to FI, it is not clear how children’s interpretation of household financial management strategies relates to this subconstruct.

#### 3.5.3. Social Identity and Comparison to Others

Social comparison was a key aspect of how children could recognize/identify instances of FI in their social environment, particularly in school settings:

“Because like all the other kids, when you look in their lunchboxes at school they have a lot of food and good food but then it’s like you only have like four, three, three or four things, and they have like five or six.”

(Lily, 9 F)

“Kids come to school without any food. Like no lunch.”

(Freya, 10 F)

Being identified as not having enough food was seen by children as potentially stigmatizing and something to be avoided, “Because they don’t want people to think that their family is really poor.” (Sundew, 9 F), and associated with negative emotional consequences:

“Maybe someone’s like said something to them in the past and bring something up about like, oh, you don’t have enough food. Or like their lunch is like very small. Someone has said something about that and that can make them feel like embarrassed or self-conscious”

(Peach, 12 F)

The threat of bullying or teasing because of a child’s food situation was also noted: “Maybe, um, there’s lots of bullies around the school, so they’re scared to show that they have no food.” (Ruby, 8 F); “Because like those guys, like they’ll get teased.” (Justin, 8 M).

One child (Justin, 8 M) was very clear in highlighting the importance of context in experiencing emotional consequences related to being identified as not having enough food or money in discussing CFIES items eight and nine:

[Item 8] Mandy: Did you feel embarrassed or ashamed because your family didn’t have enough food?

Justin: I would be, if it’s in public, because like people are always around, stalkers. But at home, like, you can feel safe…But if it’s like outside, outside world. It would feel bad all of the times…

[Item 9] Justin (joins in reading the question): So did you feel sad or mad because your family didn’t have enough food?

Mandy: Yeah. Yeah. Is that easy to understand this question?

Justin: Not really.

Mandy: No? What makes it hard do you think?

Justin: Just, like the question.

Mandy: The question?

Justin: Yes, because like, especially on the public or inner space, so it could be like your space or like outer space as in the public.

Mandy: So that’s a different thing? So it can kind of like mean different things if it’s kind of in a private or public kind of space?

Justin: Public, you would feel a bit sad or mad, but if it was like home, like, if you have any tips like, if you had $3 or $4, you could give it to your parents. They could get the food. Or if you’re, like, about 18 or 20, then you can go to get the food.

This exchange details how Justin understands the difference between one’s social identity in a “front-stage” or public setting versus “back-stage” or private/home setting in influencing what emotional responses might arise. He includes in his discussion the concept of a child initiating efforts to make resources stretch or to assist caregivers in obtaining food.

Overall, children displayed nuanced understandings of what it means to be identified as not having enough money or food, particularly in relevant social contexts such as school settings, and could describe the emotional consequences of this and associated high stakes implications for a child’s social identity. These findings are relevant when considering the CFIES items and children’s willingness to retrieve and report such experiences in a survey context perceived to be “emotionally safe”.

#### 3.5.4. Caregivers Doing Their Best and Making Sacrifices for Their Children

Participating children also prominently spoke about their parents/caregivers as doing their best and making sacrifices for their children. There was recognition of caregivers as working hard, sometimes for little reward: “I’ve been really understanding how hard my mum and Peter, my stepdad, work, so that they can afford the rent and the food money.” (Romie, 12 F). One child offered a story about why a child might feel sad or mad about not having enough food that recognizes parental sacrifice (within wider macroeconomic conditions):

“They might be feeling sad. So this is a Jerry’s dad *using a robot prop from detective kit* and he works a long time in a power plant working at his controller and he works all during the night… and then he doesn’t earn enough money. So then Jerry feels mad at where his dad works, because they’re not getting enough money to buy food and pay the taxes.”

(Kevin, 9 M)

Children recognized caregivers as primarily prioritizing their children over themselves. Scarlett spoke about meal occasions with her mother providing her favorite cut of meat to her children (though not in the context of FI):

“And with my mom she’s like “Oh yes! I have this meat again”. And then she’s like, “Oh, I’ve been at work all day”. And then she’s like, “Oh, I should give it to the kids” and then she gives it to us. Then sometimes I feel like my mom and dad’s been putting too much care into me and Mikey than themselves.”

(Scarlett, 8 F)

Within the context of FI, Romie (12 F) recounted: “I did feel ashamed when I was younger and I noticed that my mum wouldn’t eat because she was, well she didn’t have enough money to buy her own food.”

As detailed in Section 3.2.1, caregiver–child protection dynamics were apparent within the interview response process when an active caregiver was present. However, these dynamics also featured in discussions where children recognized the burden their caregivers might carry in the context of FI more broadly and that this often resulted in children wanting to present their caregivers favorably, “I felt bad for Mummy that she was trying to do her best with all the money she could have…Mummy does it the best at all of them though, because mummy is the best mummy.” (Sophia, 9 F); or as evidenced in this exchange between Tom, his mum and Mandy where he offered a potential reason why a child might feel embarrassed about not having enough food but wanted to stress this situation did not apply to his own mother:

Tom’s mum: Ah, you mean, it would be embarrassing if mum came home with beer, but not with food?

Tom: But she never, she never drinks beer, though. She never drinks beer though.

Mandy: Yeah, yeah. But thinking like, for other kids that you, in your head, you’re thinking maybe if they came home with beer or something, rather than food, that might be embarrassing for another kid, maybe?

Tom: That would be so embarrassing. But that never happened because my mum never drank beer in her life.

These findings may impact if children judge they can report their experiences of FI so as not to implicate their caregiver as failing in their role. Bart (12 M) suggests this as a reason why a child may not feel comfortable in providing a response to CFIES item two “worrying about how hard it is for their parents to get enough food for their family”: “Mmm, maybe if they think that their parents can’t do it, like, can’t get it. So they think, oh, I don’t really wanna…”. While this judgement may apply to specific CFIES items as per above, it may also impact on children’s overall willingness to engage with items that potentially threaten their caregiver.

### 3.6. Children’s Overall Impressions of CFIES

When asked to provide an overall impression of the CFIES items, children generally responded positively: “I think they are pretty good” (Bob, 12 M); “I’d say that they’re, like, good and they’re understandable and they make sense.” (Bart, 12 M); “I think they’re good questions.” (Freya, 10 F); “Yeah, I don’t think a lot of them [children] would have trouble or anything.” (Ruby, 8 F). However, some children viewed the ten CFIES items as repetitive: “They’re saying the same thing. But they use different words for it.” (Sophia, 8 F); “They’re all the same.” (Bethany, 11 F); “I think some are a bit similar to others.” (Bart, 12 M). The perceived repetitiveness was seen by some children as potentially “tiring” (Bethany, 11 F) and annoying:

Mandy: Do you think these questions are okay to ask kids?

Bethany: I don’t see why not, but it might annoy them that they’re just repetitive.

Children gave a variety of responses when asked what they thought the CFIES items were asking about generally, with most identifying the questions as centered around the family food situation and the economic access: “Um, like the situation of your, um, your family’s situation of food.” (Bob, 12 M); “Um, like, if you feel like your family needs more money to pay for things” (Kevin, 9 M); “Um, asking how much, how much, how much you should spend and how much you should not spend.” (Ruby, 8 F); “If anything, if like any type of food didn’t have, didn’t like get or have enough. But also about the emotions. So about like the embarrassed. Yeah. Whatever that one is…that looks like frustrated or something.” (Justin, 8 M); “It’s like, trying to see what, like, you think is going on with the food situation in your household.” (Bart, 12 M).

Some children assessed the overall CFIES questions as simple and easy to understand, though others felt the questions were perhaps more “medium” (Strawberry, 9 F) in terms of degree of difficulty: “Questions that I didn’t find extremely hard to find, or extremely easy to find. But questions that, like, had to put some effort into it.” (Scarlett, 9 F). Other children identified that certain items were more challenging than others to answer (see Section 3.3.3 above for item-specific findings regarding comprehension).

Mandy: A bit easy? So you found it easy? Nothing too hard to think about when answering them?

Lily: No, not really. Sometimes. Yeah, like a bit of the questions, but not…like a couple [of questions].

Children perceived younger children as potentially having more difficulty: “Like if you gave them to like, someone like younger, they would be like, maybe need their parents help. It’s like, yeah, they don’t know how to word it properly.” (Peach, 12 F); “I think anyone would understand it. But like the kids under four, maybe under four or five, maybe not.” (Mikey, 10 M).

Children reported varying responses to how exploring the CFIES questions in this interview setting made them feel/think. For some children without FI experiences, this prompted thoughts about other “Well…it sort of made me think about the, about what might be happening in other families, or like in other places” (Mikey, 10 M). This sometimes resulted in an emotional response: “It sort of makes me realize a lot more…like of like food. And it makes me feel a lot more grateful for my food.” (Bob, 12 M); “Uh, pretty sad, because some people don’t have enough food to eat.” (Kevin, 9 M). Other children, particularly those with FI experiences, cautioned the CFIES questions could be emotionally laden: “Um, yeah, they’re, they’re…I mean, they’re just questions that could be a little triggering to someone with trauma.” (Romie, 12 F); “It, it felt nice to talk about how I felt. But also I don’t want to ask anyone else about it, I don’t want anyone else to know about it.” (Sophia, 9 F).

### 3.7. When Is It Appropriate to Ask Children About FI?

Children felt, in spite of potential sensitivity or discomfort discussing the CFIES items, this would be warranted if the information gathered was to help them and other children: “I reckon it’s okay, because, like, you’re gathering it to get more like information about this type of stuff. So like helping things.” (Bart, 12 M); “If you’re researching or trying to invest in them or their wellbeing.” (Bethany, 11 F).

Children cautioned that the purpose of gathering information on children’s FI experiences needed to be benevolent: “That would be okay if it was like trying to help them but not if the questions… if they just want to be mean, that’s different.” (Sundew, 9 F).

Also, children emphasized it was necessary to be clear about the purpose for asking such questions and being transparent about how this child-reported data will be used. The following quote from Romie discusses her past experiences of providing child-report survey responses on a different topic (teachers in school):

“I don’t even know if it does anything. Because, in the survey one time…it was, how much do you think the teachers care about you? And I kept putting one for everything because, like, that’s just how I felt. And then, I don’t think it made a difference…It made me feel like a little stupid because I gave my information to people and then I didn’t even know if they did anything with it. So it kind of felt like I was exposed.”

(Romie, 12 F)

## 4. Discussion

This study used adapted qualitative cognitive interview techniques with eight-to-12-year-old children to explore the face and content validity of the recently developed 10-item CFIES tool. It provides much needed child-reported data regarding their comprehension and understanding of the measure as well as their sophisticated and nuanced insights into wider contextual considerations, which may influence response processes when engaging with the measure. The study found that within the Australian context there were challenges with comprehension of some CFIES items. The reference period of 12 months and the response categories were particularly problematic.

### 4.1. Children’s Understanding and Comprehension

This research confirms that children are aware of FI, not just in their own households but also in their wider community and can report on the potential indicators and implications. Children had a sophisticated understanding of the social ramifications of FI and identified that deployment of the tool in certain contexts needs to be considered carefully. This aligns with the findings of a recent review that children’s decision-making processes are geared towards maintaining social standing and avoiding negative consequences [20]. Even though only a minority of households reported current FI and no children reported very low food security, children showed recognition of potential reasons for FI and showed insight and empathy for children who may experience FI. They were also aware of children’s attempts to hide FI or to employ strategies to improve the family’s food situation even though they may not have employed these strategies themselves. This research therefore demonstrates children’s understandings of FI as congruent with the conceptual model underpinning the CFIES—awareness of FI across cognitive, physical and emotional domains, as well as their potential agency in initiating efforts to make food resources stretch [22]. In the Australian context this aligns with work undertaken with children aged 10–13 experiencing disadvantage, who were able to articulate the characteristics and consequences of FI and noted children as active agents in shaping and making sense of their lives [17]. They were able to distinguish physiological hunger from hunger associated with FI.

The first key challenge identified in this study was the apparent difficulty for children in applying the 12-month reference period and response options to their experiences. Cognitive testing for the closely related CFSA measure resulted in a change to the original one-month time reference to 12 months because the authors found children generally couched their food-related experiences in relation to holidays, seasons and time of the year [27]. This may provide insight into how the reference timeframe may be better contextualized. In our study, children used significant events, holidays and place-based memories to facilitate their memory of the past year when probed. However, an uncontextualized “in the last 12 months” instruction may not be sufficient to help children in their responses. While the exact phrasing of the reference timeframe would be dependent on the overall survey purpose and context, there are instructive examples demonstrating how specific and contextual time anchors can be used [31,43,44].

A recent review appraised recall periods in self-reported health surveys among children. Review findings indicated shorter recall periods were preferable. Children over the age of eight could use 7-day and 4-week recall periods, and children aged 13–18 preferred 24 h but could remember a one-month time frame, though this was linked to clinic appointments [45]. FI is a different concept, but given the cognitive difficulties in conceptualizing beyond a maximum of a month without a time anchor, the 12-month timeframe may not be appropriate.

Additionally, some children who had FI experiences out of the 12-month timeframe displayed a keenness to share these within the interview. This tendency has been found in literature looking at survey practice in non-survey literate adult populations. Massey [46] found survey respondents were eager to share the full details of their lives rather than just the specific information requested by the survey, which could be seen as reflecting a deeper need for respondents to have their broader experiences acknowledged, while impacting efficiency of survey administration and potentially validity. A content validity study of a child abuse screening tool in adolescents included a recommendation to add extra writing space for participants to share experiences/contextualize their responses based on similar findings of willingness to share detailed experiences in the interview context [47]. Nonetheless, the degree which children are willing to engage in sharing experiences will be impacted by their perception of the safety of the survey context and also their beliefs about how such disclosures may be treated within the context. The findings also indicated that how the interview is conducted and who is present is important with respect to how children respond.

### 4.2. Impacts on Survey Response: Question Sensitivity and Further Contextual Considerations Identified by Children

Overall, while the topics for CFIES questions may be relevant to the construct of FI, the salience of the emotional and social aspects of FI experiences to participants meant they identified that careful consideration needs to be given to how, where and with whom the survey is deployed. Survey modality has been previously considered as a potential impact on responses. Whether a survey is deployed face-to-face, via phone or is web-based can introduce interviewer effects and social desirability bias [48]. Survey modality may not matter for generic questions but can be much greater for questions that are considered sensitive (such as those around FI) [49]. Survey modality has been considered as important or as a key factor in many validation studies.

All interviews for this study were face-to-face with the same interviewer, and consequently the impact of different survey modalities could not be assessed. However, the presence and involvement of the caregiver in the interview varied. The presence of caregivers in this specific interview setting influenced children’s involvement. Children actively seeking input from their caregiver may have indicated difficulty in or lack of understanding regarding some items. Presence of caregivers can change child responses due to social desirability bias, the need to protect caregivers or family privacy and reluctance to disclose risky behaviors [50]. The drive to maintain social identity for both adults and children [17] can mean that responding to FI questions is a morally contested experience and cannot be undertaken lightly [51,52].

The nuanced contextual considerations offered by children have significance for how the CFIES items are interpreted and whether relevant experiences are retrieved. For example, children identified hunger as a highly recognizable and undesirable physiological state. This high salience of “physiological hunger” to children could overshadow aspects of the CFIES item, which qualifies hunger as a result of lacking access to “enough food”. This may also impact the timeframe of reporting, since even events in the past seem highly salient, making it hard to disentangle whether experiences of FI are past or current.

### 4.3. Key Aspects of Validity to Consider Prior to Using CFIES—Reflections from an Australian Setting with Children Aged 8 to 12 Years

Face and content validity testing, particularly using cognitive interview techniques, is often a poorly reported component of measure development [24]. In the case of the CFIES, this measure was developed by drawing upon extensive international qualitative work with children to develop child-report FI measures in three separate settings [27,28,31]. These measures sought children’s perspectives using extensive concept elicitation work [13,16,53]. However, details regarding the pilot testing/cognitive interviewing of these three measures and the CFIES itself are lacking. For the CFIES, its pilot study involved deployment in a large global sample across economic contexts and a wide age range. Internal consistency, cross-cultural measurement invariance, as well as criterion validity were examined in this large sample with promising results. However, despite this, only a small group (N = 8) of Canadian 10-year-olds were involved in cognitive interviewing to evaluate their understanding and improve wording of the items, with minimal reporting of this process and its outcomes [32]. Consensus-based quality standards in the related field of patient-reported outcome measure (PROM) development suggests there is increased risk of bias for measures if the tool’s comprehensiveness, comprehensibility and relevance in the target population is not explored or reported [23,24]. In Frongillo et al. [32], several survey modalities were deployed in its pilot survey and were not perceived to significantly impact measure validity. This is despite the extensive work that details shame and stigma surrounding FI experiences for families including children [29,54]. It is also in contrast to work in other disciplines examining child-report and the impact of survey modality on survey response [45]. Our findings suggest that children who have had FI experiences may feel uncomfortable about revealing such information if the context is not appropriate and the reasons for data collection are not clear. Developing an enhanced self-administered digital version of the CFIES may be one way to reduce discomfort and enhance privacy (see Section 4.5 for further discussion of this). As stated earlier, the difficulty of children to conceptualize and recall experiences based on the 12-month scale suggests it may be preferable to modify to a shorter recall period or employ temporal anchors based on recent holidays or memorable events. The response categories were also found to convey varied meanings for different children. Therefore, the use of simpler response categories (e.g., binary responses such as yes/no) may be preferable, though further testing of these would be warranted. Words causing confusion such as “guardians” and “limited” may need to be replaced with more direct child-derived descriptions. Our findings broadly support the use of the tool within an Australian setting with 8-to-12-year-olds, though they highlight key contextual factors to consider and indicate areas where potential adaptions may enhance validity.

### 4.4. Strengths and Limitations

A major strength of this study is its prioritization of children’s perspectives within a process of detailed investigation into and reporting of the face and content validity of a recently developed child-report FI measure. These validity properties are often overlooked and underreported yet arguably underpin all other psychometric properties [24]. Going beyond merely troubleshooting “mechanical” measure issues, cognitive interviewing techniques may serve to provide illustrative detail about the various phenomena captured by a question and, ultimately, represented in a survey statistic [42]. The study contributes to an international body of work relating to the important issue of children’s FI, from a novel Australian setting. The potential threats to validity identified in this interview study, while not generalizable given the small sample and high-income context, provide the basis for further exploration of potential issues in other populations. The use of an eight-to-12-year-old age range is helpful as the original pilot study was characterized by a slightly older sample [32]. Therefore, this study may provide particularly useful insights for those looking to use the CFIES in eight-to-12-year-olds and may particularly highlight areas of caution to look at in those younger than eight years. It is also important to recognize that there may be different experiences of FI by children within the same household, particularly across age categories, with older children shielding younger siblings by giving their own foods, as an example [14,16]. Further research is warranted to support detailed guidance regarding which child is selected to report FI experiences within a household when using the CFIES.

Though diversity in sampling was an aim of recruitment, families tended to be on higher incomes with caregivers with higher levels of education. There were a range of cultural backgrounds included in the study, including families with migration experiences. However, no Indigenous families were recruited. Many Indigenous families have experienced structural violence and racism at the behest of invading colonizers, including the use of food as a weapon, child removal and family/community deconstruction [54,55]. Consequently, Indigenous families may have more at stake in responding to these questions, and any measure should not be assumed as culturally safe in these contexts [56]. Further testing of the cultural appropriateness of these items or the development of a different modality, particularly for children from First Nations’ backgrounds, will be required with additional ethical and cultural considerations. This work is particularly pressing given that Indigenous families are disproportionately affected by FI, resulting in significant health disparities [57]. Exploration of cultural considerations should be done with Indigenous investigators, employing culturally appropriate methodology, which was not within scope for this study. Similarly, households in the study were generally of a smaller size with no extended or multigenerational co-habiting families included. Blended families were present within the sample (as identified in research onboarding or within the interview itself) but were not specifically measured in the sociodemographic survey, nor was the experience of coming from a “blended family” specifically probed. This may have implications for the level of detail gained from the sample regarding concepts like “family” or wider intrahousehold dynamics around food and warrants further investigation. Our sample also included several children on medications impacting appetite (both suppressant and inducing), mainly to treat ADHD. Our findings indicate that these children may have particularly heightened perceptions of hunger or may go for extended periods without food and therefore may interpret questions in light of these experiences. Potential users of the tool should consider the prevalence of conditions like ADHD and medication use in their respective context to aid interpretation efforts.

While 33% of families reported some experience of food insecurity ever, few families reported current child-level FI, particularly at the severe level. The final sample included families of middle- to high-income, and caregivers tended to have tertiary education backgrounds, indicating potential selection bias. Social identity issues and the threat of stigmatization may have impacted families’ comfort in signing up to such a study and may have prevented families with severe FI experiences from participating. Attempts to ameliorate potential stigma included using thoughtful wording in participant information material and by partnering with trusted organizations working with families at high risk of FI to distribute recruitment materials. However, this study may have benefited from greater personal relationship building and outreach efforts through a more extensive pre-engagement process. Authentic reciprocity to facilitate social connections and engender trust is crucial to build engagement with service providers/gatekeepers who may facilitate access to vulnerable families for inclusion in research [58]. Further studies inclusive of those with more severe FI experiences may help to identify further pertinent issues with the tool and extend this study’s findings.

Furthermore, while this study sought children’s perspectives and attempted to center these in detailed face and content validity investigation of a child-centered measure, children were not involved in research design or data analysis stages. A small pilot study sought children’s perspectives on the interview guide, consent and participant information materials and recruitment collateral and changes were made based on this feedback. However, child engagement earlier in study design may have resulted in a study more in alignment which children’s priorities [59].

### 4.5. Implications for Research and Practice

The CFIES is a recently developed child-report tool that draws upon qualitative work with children across economic contexts. Despite displaying promising psychometric properties in a recent pilot global study, there is a lack of detailed reporting of face and content validity testing with members of its target sample. Despite its potential, it should be employed and interpreted with some caution given this study’s findings. This is particularly important if the measure is adapted for use outside of the research context. The value of global FI monitoring measures for advocacy purposes has been noted [2], and the consistent and routine use of measures in certain settings, namely North America, has enabled a significant body of work to be established looking at determinants, outcomes and ways to evaluate FI interventions [60]. The inherent tension in developing measures for local requirements without compromising comparability or validity has long been a feature of commentary within the FI field [61,62]. Brief, simple and standardized tools are often popular given their perceived ease of use, ability to be embedded in larger related survey instruments and contribution to a shared body of evidence. Yet, the establishment of systematic guidelines (like COSMIN) demonstrates the drive to improve the quality of self-report measures by particularly recognizing the value of including participant lived experiences as a key component of content validity. Any potential users of a measure need to consider these competing priorities and efforts to understand the validity of a tool within a context of interest are likely to be instructive. Adapting the CFIES for use in practice settings outside of research may extend the number of data systems available to gather child-reported FI, though any adaptation initiatives would require further investigation.

Nonetheless, there may be ways to enhance content and face validity and respond to potential issues without wholesale adaptation of items requiring further extensive validation initiatives resulting in multiple survey versions. For instance, probes that might reorient the respondent to the salient part of the question may help to ensure responses are in alignment with question designer intent. Using exemplars from concept elicitation work with the target population or use of highlighting or moving the most salient part of an item to the front of the question may help facilitate the response process [47]. Elements may be used in self-administered digital versions of the measure so children can access help to explain confusing words or parts of items. This may be in the form of a “help” button providing child-derived advice or suggestions in audio and/or visual formats. Similar elements may be used to help support perceptions of safety and comfort in a digital context such as virtual “stop signs” or “safe exit” buttons as well as virtual fidgets or sensory elements/widgets.

In practice, for interviewer-administered modalities, careful consideration needs to be given regarding the presence of caregivers and their involvement. All the caregivers in this study were supportive, though this may not be the case in practice where children may be fearful of responding in ways that may elicit a negative response. Negotiating caregiver involvement in both research and practice settings needs careful consideration by any potential users of this tool, given the possibility for caregivers to assist their children in tool completion versus negatively influencing their response. Children ethically need to be aware of the purpose of data collection as the purpose may influence how they respond and ideally need to be given the opportunity to decide about the presence or absence of caregivers.

## 5. Conclusions

Child-report FI measures provide a way for children’s unique perspectives and experiences to be foregrounded when investigating the significant public health and social issue of food insecurity. Nonetheless, the context a tool is used within matters, and this needs to be considered when applying or adapting the CFIES to specific settings and purposes. This study broadly supports face and content validity of the measure in eight-to 12-year-olds but highlights potential limitations and shortcomings, particularly from children’s perspectives. This includes children’s challenges in applying a 12-month time reference and choosing from the available response options. Child–caregiver protection dynamics and the threats to social identity and potential for stigma, as highlighted in this study and congruent with other qualitative child FI literature, may play a significant role in how children choose to respond to the CFIES items and need to be deeply considered by tool users.

A global monitoring tool that reflects broad cross-cultural understandings and conceptualization of FI from children’s perspectives represents a feasible and pragmatic way to collect data that is useful in tracking change in FI over time across regions and may have an important role to play in advocacy efforts. However, this study suggests building rapport with communities and organizations that allow for the interpretation of the data within the contextual considerations is necessary. Adapting the CFIES to service delivery/practice settings may provide a way to further understand and respond to children’s FI experiences in their daily lives, though this may require significant additional work, and may not be feasible. Recognizing the limitations and validity of the tool while supporting children’s engagement and understanding of its purpose may contribute to more contextual, nuanced interpretation of children’s FI experiences, which may better inform potential solutions.

## Figures and Tables

**Table 1:** Child Food Insecurity Experiences Scale items.

For each question, please answer whether it happened many times, 1–2 times, or never in the past 12 months.
In the last 12 months … 1. Did you worry that food at home would run out before your family was able to get more? 2. Did you worry about how hard it is for your parents/guardians to get enough food for your family? 3. Were you not able to get the food you wanted because there was not enough money? 4. Has the size of your meal been cut because your family did not have enough food? 5. Were you hungry but did not eat because your family did not have enough food? 6. Did you skip a meal because your family did not have enough food? 7. Did you feel tired or weak because your family did not have enough food to eat? 8. Did you feel embarrassed or ashamed because your family did not have enough food? 9. Did you feel sad or mad because your family did not have enough food? 10. Did you feel embarrassed or ashamed about any of the things you or your family had to do to get enough food? Responses and scoring for each question: Many times (scored 2); 1 or 2 times (scored 1); never (scored 0); do not know; or refuse to answer A total score can be calculated by summing individual item scores (0–20) categorized into: no food insecurity experience (a score of 0), few experiences (a score of 1 to 6), several food insecurity experiences (a score of 7 to 10), and many experiences (a score of 11 or more).

**Table 2:** Overall sociodemographic characteristics of children (N = 25), their caregivers and their household (N = 17).

Characteristic	Total
**Participating child characteristics**	
Age (y) (mean, range)	
Age	9.6 (8–12)
Sex (%, *n*)	
Girl	48 (12)
Boy	52 (13)
Diagnosed medical or mental health condition that affects eating or appetite, as reported by caregiver * (%, *n*)	
Yes	19 (4)
No	81 (17)
Missing	4
Developmental concerns for child/children, as reported by caregiver (%, *n*)	
Yes	19 (4)
No	81 (17)
Missing	4
**Household and caregiver characteristics**	
Highest level of education completed by caregiver (%, *n*)	
Year 11 or Year 12 equivalent	6 (1)
TAFE/technical college or trade certificate/apprenticeship	18 (3)
Undergraduate university degree	18 (3)
Postgraduate university degree	47 (8)
Missing	12 (2)
Size of household	
# of people (mean, range)	4.2 (3–6)
# of children	1.8 (1–3)
Annual family income (per week) (AUD) (%, *n*)	
$0–$25,999 ($0–$499 per week)	6 (1)
$52,000–$103,999 ($1000–$1999 per week)	18 (3)
$104,000–$207,999 ($2000–$3999 per week)	23.5 (4)
$208,000 + ($4000+ per week)	23.5 (4)
Do not know/prefer not to say	18 (3)
Missing	12 (2)

* Reported conditions included attention hyperactivity deficit disorder (ADHD), autism spectrum disorder (ASD), oppositional defiant disorder (ODD), anxiety and post-traumatic disorder (PTSD). Medications were reported as impacting appetite by caregivers.

**Table 3:** Food security status of households (N = 15).

Food Security (FS) Status *^	Household (18 Items) % (*n*)	Adult (10 Items) % (*n*)	Child (8 Items) % (*n)*
High FS	67 (10)	80 (12)	73 (11)
Marginal FS	7 (1)	7 (1)	20 (3)
Low FS	13 (2)	13 (2)	7 (1)
Very Low FS	13 (2)	0 (0)	0 (0)

* Data was not collected for two households; ^ definitions as per USDA HFSSM scoring protocol [7]. High FS = nil concerns about food access, Marginal FS = some level of anxiety about food access, Low FS = quality of foods available for consumption compromised, Very Low FS = quantity of foods available for consumption compromised. Food insecurity (FI) = Marginal, Low or Very Low FS.

**Table 4:** Key descriptive terms in CFIES with alternative words and interpretations provided by children.

Key Descriptive Terms in CFIES	Alternative Words and Meanings Suggested by Children
family (items 1 and 2)	**What do you think of when you hear the word family? Who do you think of?** “My dad, my mum, my sister, my brother and my cats.” (Kevin, 12 M) “People that live in your house.” (Zac, 8 M) “People who live with you and who love you.” (Peppa Pig, 11 F) “Mummy, me, Bethany [sister], Lydia [sister], Benny [pet], Boo [pet]…And sometimes other family and I don’t want to talk about other family.” (Sophia, 8 F) “I think of a happy, big family, but not everybody has that.” (Romie, 12 F) **What does the word “family” mean to you?** “Sort of means like, I don’t know, the people in families who are loving and caring…they’re always there for me.” (Sundew, 9 F)
parents/guardians (item 1)	**Parents/guardians. What do those words mean to you?** “Protectors.” (Zac, 8 M) “It means to me like people that like, like raise you and live with you.” (Strawberry, 9 F) “People who care for you and like the people who look after you.” (Lily, 9 F) **Do you have any thoughts about the word “guardian” in the question?** “Your parents are like your birth parents, your guardians might be like your adopted parents, or like people who take care of you sometimes.” (Peppa Pig, 11 F) **Reasons to include word “guardian” in item** “I think it’s good because some people might not have, like they have guardians instead.” (Starfire, 9 F) **Does including both parent/guardian make the question harder?** Romie: Not really because it’s like accepting to those people who don’t actually have parents. Mandy: Yeah, so it’s sort of accepting. Including? Romie: Inclusive, yes.
“run out of food” (item 1)	**How to tell if family has run out of food** “Um, they might, um, they might not be going out as much.” (Peppa Pig, 11 F) **Reasons for running out** “Because they can’t afford to get more food or there’s not like enough food in stock.” (Bethany, 11 F)
“foods wanted but cannot get” (item 3)	**Foods wanted but cannot get because of money** “Uh, I don’t know, maybe if I wanted eight litres of ice cream.” (Kevin, 9 M) “They would probably pick chips and snacks…not like full food.” (Freya, 10 F) “Maybe just food in general.” (Bethany, 12 F) “I mean, kids do want like chocolate and lollies. But they don’t cost a lot.” (Kevin, 9 M) **Reason for foods being restricted** “No, it’s just because mom said it’s not healthy.” (Bethany, 11 F) “Well. Some companies do make it very expensive just for chocolate. And, um, it’s just not healthy.” (Pilot, 12 M) “Not, not, not having enough money. I don’t think that’s a problem…It’s just like, cause she doesn’t want to buy them and have them in the house and like stuff like that.” (Peach, 12 F)
limited (item 4)	“rationable”, “there’s only this amount of food and there’s no more”, “smaller food”, “smaller amount”, “not much”, “hardly any”, “you can’t have more”, “down to a certain amount”, “not an ongoing source of it”, “you can only choose certain foods…you don’t get a wide choice”
“skip a meal” (item 6)	**What do you think of when you hear “skip a meal”? What do those words mean to you?** “You don’t have it. And you just don’t eat that at that time.” (Kevin, 9 M) “I think of like, not having breakfast. Or skipping lunch. Because like, you forget.” (Freya, 10 F) “Skip a meal. I don’t get it.” (Sophia, 8 F) “Well, makes me think that like you just you didn’t have breakfast and just went ran off to school.” (Ruby, 8 F) “I have skipped brekkie sometimes because I want to get to school on time. Not because my family didn’t have enough food.” (Archer, 9 M) “Like, skip is like, when somebody says, “Tom, it’s time to eat, I like say, skip. “Like, I don’t want to eat. I’m not hungry”. Like, sometimes I do that. But when I’m, when I actually don’t feel like eating, I just say, Mum, I don’t feel like eating today.” (Tom, 9 M) “Well, like just going to bed without, like having a meal. Yeah, I’ve done that before because I’ve had way too much afternoon tea. Like I’ve had a whole dinner for afternoon tea.” (Pilot, 11 M)
meal (item 4)	**Meal as constituted by multiple elements** “It’s like, not one, like, particular food. It’s like, like a burger, chips and like a drink.” (Strawberry, 9 F) “Like, a little feast…like, rice, soup, egg and meat. It’s like about four stuff.” (Justin, 8 M) “Um, the family, the plate, the glass of water, my main thing which is like a meat or something, and then some sort of veggie side, sometimes like some sort of carbohydrates, like meat, like some wedges or some chips.” (Bethany, 11 F) **Meal as one of three occasions over day** “Breakfast, lunch, dinner.” (Pilot, 11 M)
“things you or your family had to do to get enough food” (item 10)	**Can you think of anything that they would do or their family would do that would make them embarrassed or ashamed?** “Eat food off the streets because they don’t have enough food…They might like…if they don’t have any food at all, they might have to like…sneakily get food from like a shop.” (Strawberry, 9 F) “Well, they could beg and that might feel you, disappointed and embarrassed, all those… well, they could um, go to, like, St. Vinny’s De Paul and stuff like that.” (Goku, 9 M) “Um, maybe they might have ask other people for help.” (Peppa Pig, 11 F) “Yeah, it would be sort of embarrassing, like maybe to beg people to give you food. It’s probably not a nice thing to do. And no, no one would want to go through it.” (Mikey, 10 M)

**Table 5:** Key emotion words in CFIES items.

Key Emotion Word in CFIES and Emojis Chosen by Children to Represent Word	Synonyms Suggested by Children	Recognizing Emotion in Self/Others and Reasons for Feeling Emotion if Not Enough Food
worry (item 1) 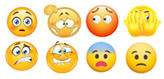	Desperate, scary/scared, stressed out, “don’t know what to do”, afraid, “something can go or be finished”, nervous, “something bad will happen”, “kind of like fear”, nervous, stress, terrified, anxious, “not confident and not 100 percent sure”, overthink	“Yeah, because they’re like, you can tell by their expression on their face.” (Strawberry, 9 F) “You can’t always tell, but sometimes you can. It depends on how they’re acting. But sometimes people are really good at hiding it.” (Romie, 12 F)
tired/weak (item 7) nil emojis identified in presented set	tired—drowsy, sleepy, “you might want to go to bed/have a big rest”, weak—sick, uneasy, “you just don’t want to do anything”, “you’re not strong at that moment”, “you can’t really carry that much weight”, “weak in energy”, “when a grown-ups not gone to the gym”, “you have no energy”, “not strong”, exhausted, “all you want to do is lay down”, “sick in the tummy”	“Like they’re not like running around at lunch.” (Bob, 12 M) “Well, if they’re tired, they’ll probably want to close their eyes, or oh, they’re probably, um, they’re probably sitting down trying to sleep.” (Ruby, 8 F) “And they just like, when they walk to a park, they just go to a park and they don’t do anything.” (Sophia, 9 F) “Not like super happy and bright and awake” Freya, 10 F **Why feel tired/weak?** “Well, um, they couldn’t have like, eaten enough, like a normal, average human body needs.” (Starfire, 9 F)
embarrassed/ashamed (items 8 and 10) 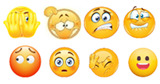 “Because you would never look like that *points to smiling emoji*. Unless you are trying to hide it like everyone says.” (Pilot, 11 M)	anxious, shy, “doesn’t want to show its face”, ”scared because like the red cheeks he doesn’t want to show it”, “you want to hide away”. “upset”, disappointed **Difference between embarrassed versus ashamed** “Maybe for ashamed, like maybe like maybe not really have like the word “ashamed” in there. Because like embarrassed mainly explains it to me.” (Starfire, 9 F) “Um, to me, they sound the same, but probably to some other people, they don’t.” (Tom, 9 M)	“They’ll probably be avoiding eye contact, they might be blushing with red cheeks.” (Kevin, 9 M) “Yes, because sometimes when my friends feel ashamed they cry.” (Zac, 8 M) “The face might go red.” (Owen, 8 M) “Scared because like the red cheeks he doesn’t want to show it.” (Justin, 8 M) **Why might feel embarrassed or ashamed?** “Because like all the other kids, when you look in their lunchboxes at school they have a lot of food and good food but then it’s like you only have like four, three, three or four things, and they have like five or six.” (Lily, 9 F) “Maybe they’re scared of other people laughing at them.” (Ruby, 8 F)
sad/mad (item 9) sad 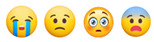 mad 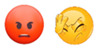	sad—worried, distraught, anxious, blue, gloomy, “when you don’t feel so good and stuff” mad—angry, “you’re really worked up and angry” **Difference between sad and mad** “They mean different things because you could be sad or you could be mad.” (Sundew, 9 F)	**Why might feel sad/mad?** “They might feel mad because they see other people with food and they feel like they’re missing out.” (Goku, 9 M) “They might feel embarrassed or ashamed because people have big birthdays and they don’t because they don’t have enough money or food to celebrate it.” (Freya, 10 F) “Because their tummy might have, like, they might have been, like, hungry.” (Owen, 8 M) “Because maybe like they aren’t, like they can’t have the food that they like usually eat and they feel sad or like mad because they didn’t get what they want.” (Starfire, 9 F)

**Table 6:** Alternative wording for selected CFIES items as suggested by children.

Item	Child’s Suggested Alternative Wording of Selected CFIES Items
1 “worry food run out”	“I think if it was for, like, younger kids you could go, “Do you ever…do you ever get upset because Mummy and Daddy don’t have enough food?”” (Romie, 12 F) “Did you think that food would run out at home?” (Bart, 12 M)
2 “worry how hard it is for parents/guardians to get enough food”	“Do you worry about your parents not getting enough food for your family?” (Lily, 9 F) “Have you worried about how hard it is to get food for your family?”(Bob, 12 M)
4 “size of meal limited”	“Did you have to make the food smaller ‘cause your family didn’t have enough food?” (Lily, 9 F)
10 “embarrassed/ashamed about things had to do to get enough food”	“Did you feel scared or frightened about any of those things you or your family had to do to get enough food?” (Justin, 8 M)

## Data Availability

Summary data (coding framework) that support the findings of this study are available on request from the corresponding author. The data are not publicly available due to privacy or ethical restrictions.

## Supplementary Materials

The following supporting information can be downloaded at https://www.mdpi.com/article/10.3390/nu18142303/s1, File S1 (Table S1): COREQ (COnsolidated criteria for REporting Qualitative research) Checklist; Table S2: Exemplar probes, prompts and activity for CFIES item 1.

## Abbreviations

The following abbreviations are used in this manuscript:

ACFSS	Arab Child Food Security Scale
CFIES	Child Food Insecurity Experiences Scale
CFSA	Child Food Security Assessment
CFSSM	Child Food Security Survey Module
COSMIN	COnsensus-based Standards for the selection of health Measurement INstruments
FI	Food insecurity
FIES	Food Insecurity Experiences Scale
FNS	Food and nutrition security
HFSSM	Household Food Security Survey Module
USDA	United States Department of Agriculture
