# Supplementary material for: “That Would Give the Kids a Little Challenge to the Brain!” Co-Investigating the Child Food Insecurity Experiences Scale (CFIES) with School Aged Children: A Qualitative Cognitive Interview Study
Source: Nutrients. 2026 Jul 14;18(14):2303. doi: 10.3390/nu18142303 (PMC13416180; doi:10.3390/nu18142303)
Supplement: Supplementary file 1 [file nutrients-18-02303-s001.zip › SUPP_Supplementary table S2.pdf]

Supplementary table S2: Exemplar probes, prompts and activity for CFIES item 1

Item	Examples
CFIES item 1	Did you worry that food at home would run out before your family was able to get more?
Prompts/probes (note, not all prompts/probes used in each interview)	What do the words 'run out of food' mean to you? How would you know if someone had run out of food at home?<br>What does the word 'family' mean to you? Who do you count in your family?<br>What does the word worry mean to you? What other words mean 'worry'?<br>How does your face look if you are [EMOTION]?<br>How do you move if you are [EMOTION]?<br>How do you move your arms or legs or head or hands when you are [EMOTION]?<br>How do you know if someone is [EMOTION]?<br>What sounds would you make if you were feeling [EMOTION]?<br>If someone was [EMOTION], how would they act?<br>Is there anything confusing or hard to understand about this question?<br>Tell me what you think this question is asking, in your own words?<br>Was there anything you think kids your age would find confusing or hard to understand about this question?<br>If you were to ask kids your own age this question, is there any way you would do it differently?<br>Where do families get more food when they run out?<br>How would you know if someone had run out of food?<br>Is there anything about this question you think might mean that kids would not want to answer it? Or make them feel uncomfortable/feel bad?
Example activity	Can you show me if any of these emojis look worried to you? (use supplied emoji gallery)
